# Comparison of postoperative outcomes between bikini-incision via direct anterior approach and posterolateral approach in simultaneous bilateral total hip arthroplasty: a randomized controlled trial

**DOI:** 10.1038/s41598-023-29146-2

**Published:** 2023-04-29

**Authors:** Xin Jin, Guo Chen, Mengcun Chen, Muhammad N. Riaz, Jing Wang, Shuhua Yang, Weihua Xu

**Affiliations:** Department of Orthopaedics, Union Hospital, Tongji Medical College, Huazhong University of Science and Technology, 1277 Jiefang Avenue, Wuhan, 430030 People’s Republic of China

**Keywords:** Reconstruction, Surgery

## Abstract

The purpose of this study was to compare an oblique bikini-incision via direct anterior approach (BI-DAA) to a conventional posterolateral approach (PLA) during simultaneous bilateral total hip arthroplasty (simBTHA) in terms of early patient outcomes, postoperative functional recovery, and complications. From January 2017 to January 2020, 106 patients receiving simBTHA were enrolled and randomly allocated to the BI-DAA or PLA group. Primary outcomes were measured using hemoglobin (HGB) drop, transfusion rate, the length of stay (LOS), the visual analog scale (VAS) for pain, the Harris hip score, Western Ontario and McMaster Universities Osteoarthritis Index, and the scar cosmesis assessment and rating scale. Secondary outcomes were the operative time, radiographic measurements, including femoral offset, femoral anteversion, stem varus/valgus angle, and leg length discrepancy (LLD). The occurrence of postoperative complications was also recorded. There were no differences in demographic or clinical characteristics before surgery. Compared to the PLA, the patients in the BI-DAA group had lower HGB drop (24.7 ± 13.3 g/L vs. 34.7 ± 16.7, *P* < .01) and transfusion rates (9/50 vs. 18/50, *P* = .04) and a shorter LOS (5.12 ± 1.5 vs. 6.40 ± 2.0 days, *P* < .01) without increasing the operative time (169.7 ± 17.3 vs. 167.5 ± 21.8 min, *P* = .58). The BI-DAA group yielded a smaller LLD (2.1 ± 2.3 vs. 3.8 ± 3.0 mm, *P* < .01) and less variability in component orientation than the PLA group (100% vs. 93%, *P* = .01). As for the scar, the BI-DAA group produced a shorter incision length (9.7 ± 1.6 vs. 10.8 ± 2.0 mm, *P* < .01) and higher postoperative recovery satisfaction than the PLA group. Furthermore, the BI-DAA group had a reduced VAS score one week after surgery and had better functional recovery in three months postoperatively. The BI-DAA group had a higher incidence of LFCN dysesthesia (12/100 vs. 0/100 thighs, *P* < .01), while other complications did not differ significantly between the two groups. For simBTHA, the bikini incision offers early recovery, less variance in components orientation, better postoperative outcomes, and scar healing than the PLA. Therefore, the bikini incision could be a safe and feasible option for simBTHA recipients.

## Introduction

Total hip arthroplasty (THA), one of the most successful and frequently performed elective surgeries, can effectively relieve hip pain, significantly improve function, and improve quality of life^1,2^. For some patients with bilateral end-stage joint disease, simultaneous bilateral THA (simBTHA) is a reliable option. Several studies show that there are no differences in mortality, re-admission rates, component positioning, implant subsidence, or the time to return to sports after simBTHA compared with staged bilateral THA^3^. Moreover, simBTHA does have some potential benefits in shortening the length of stay (LOS) and reducing the costs of hospitalization, which are extremely important for younger patients with a strong preference for one-stage surgery^4^. In addition, strategies to further reduce the surgical burden of simBTHA have been discussed in some previous studies^5–7^.

Over the past decade, the direct anterior approach (DAA) has received increasing attention. Interest in surgical approach to THA continues to be high. Specific pros and cons of the DAA vs PLA have been debated in the literature with no clear consensus^8–10^. In principle, the DAA is an anatomic approach in which neuromuscular interval is used to minimize soft-tissue damage significantly^11,12^. Compared to the traditional technique, several studies have demonstrated that the DAA can reduce bleeding and discomfort, speed up recovery, and enhance functional results. It is linked to a lower risk of dislocation due to improved preservation of the posterior soft-tissue envelope, which results in faster postoperative recovery and shorter hospital stays^11,13,14^.

A modified minimal oblique incision using the groin cleavage line (bikini incision) for the DAA has recently demonstrated encouraging results, especially in improved cosmesis and postoperative scarring^13,15–17^. A study including 964 patients undergoing the bikini incision via DAA (BI-DAA) for THA showed that the bikini incision has additional advantages in curbing wound complications and improved cosmesis outcomes compared with the traditional DAA^15^.

To the best of our knowledge, no prospective RCT studies have been conducted to evaluate the early postoperative outcomes of the bikini incision for simBTHA. Furthermore, well-designed prospective randomized trials comparing the bikini incision and the posterolateral approach (PLA) for simBTHA in terms of postoperative recovery, components placement, and scar recovery satisfaction were absent in the literature. Therefore, we conducted a prospective randomized controlled trial (RCT) to compare perioperative and early postoperative outcomes between the bikini incision and the PLA among simBTHA patients. We hypothesized that bikini incision could enhance the early recovery, the accuracy of prosthesis orientation, early postoperative outcomes, and scar healing.

## Materials and methods

### Patients

**Figure 1:**
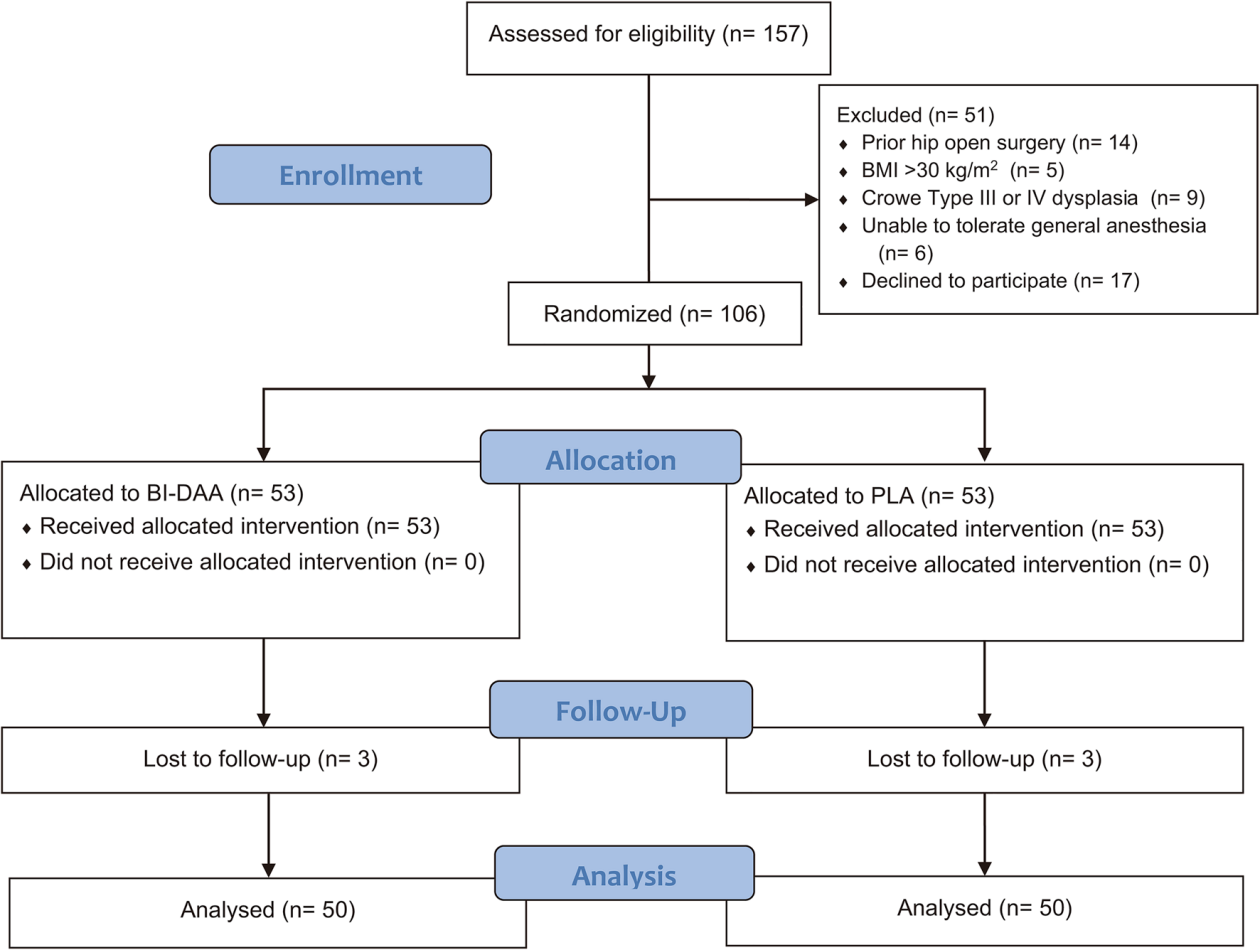
Flow diagram illustrating patient selection, randomization, follow-up, and data analysis throughout the study. BI-DAA, bikini-incision via direct anterior approach; PLA, posterolateral approach.

The study was approved by the Ethics Committee of Tongji Medical College, Huazhong University of Science and Technology (2016-S247) and registered in the Chinese Clinical Trial Registry (ChiCTR2000031041, 21/3/2020). All research was performed in accordance with the Declaration of Helsinki and relevant guidelines/regulations. All patients provided written informed consent before participating. Patients undergoing primary bilateral THA at our institution were screened between January 2017 and January 2020. The inclusion criteria were bilateral end-stage osteoarthritis of the hip, bilateral osteonecrosis of the femoral head (ONFH) (Ficat III or IV), or bilateral Crowe Type I or II dysplasia. The exclusion criteria were: (1) unilateral hip disease; (2) prior hip open surgery; (3) body mass index (BMI) > 30 kg/m^2^; (4) an inability to tolerate general anaesthesia; or (5) an unwillingness to participate in the trial. All patients were randomized into PLA or BI-DAA group using a computer-generated list of numbers. The numbers were then sealed in opaque envelopes by an investigator, who asked patients to select an envelope on the morning of their surgery. The investigator assigned the patient to a specific treatment group and then informed the surgeon based on the selected number. The data were collected and analyzed by two independent investigators. See Fig. 1 for details.

### Surgical technique

All procedures were performed by one experienced joint surgeon experienced in performing both the DAA and the PLA, who performed more than fifty bilateral arthroplasties annually. The bikini incision was performed by the method introduced by Leunig^15^. A conventional operating table was used with the patient placed supine. The acetabular component was implanted routinely based on the transverse acetabular ligament, as described by Archbold et al.^18^. To complete mobilization of the proximal femur, the capsule on the inner side of the lateral trochanter is incised preceding this dissection dorsomedially until the obturator internus tendon is exposed. This release is performed under continuous traction using a blunt bone hook placed into the femoral medullary canal. The hip was adducted, extended, and rotated to expose the proximal femur. Broaching of the femoral canal is started and proceeds up to the appropriate size. A trial reduction is performed, and the lower limb length and offset are checked manually and with C-arm confirmation. The trial components are removed, and the prostheses are placed with press-fit fixation. Routine closure was performed without drainage. While one assistant was closing up the wound, the surgeon started the procedure on the other hip. The standard posterolateral approach was used in the PLA group, well described in all major texts on orthopaedic surgery. It is worth mentioning that the interval between two sides in the PLA group took approximately 20 min. The PLA is well described in all major texts on orthopedic surgery, and we used a modified version of the Gibson-Moore method. The patient was placed in lateral position. After the skin and fascia above the greater trochanter were cut. The external rotation muscle was separated, and an incision was made in the hip joint capsule. The hip joint was dislocated, and the femoral neck was resected. The remaining steps were performed as described above. All subjects enrolled in the study received a Tri-lock Total Hip System femoral stem, a Pinnacle Acetabular Cup System cup, a Marathon cross-linked polyethylene liner, and a Biolox^®^ Delta femoral head, size 28, 32 or 36 mm (DePuy Synthes, Warsaw, IN, USA). All devices used are commercially available and were implanted according to approved labeling.

### Perioperative treatment

Weight-dosed cefathiamidine and tranexamic acid were administered before incision. Ropivacaine was infiltrated into the surgical site and around the joint before suturing. Both groups followed a standard postoperative rehabilitation protocol, which began the first day after surgery, and included immediate weight-bearing with a walker for two weeks.

### Primary outcomes

The haemoglobin (HGB) drop, the transfusion rate, and the LOS were recorded. The postoperative pain during active motion (hip flexion of 45°) was measured using a visual analogue scale (VAS) at 24 h, 72 h, 1 week, 4 weeks, and 3 months after surgery. Functional outcomes, including the Harris hip score (HHS) and the Western Ontario and McMaster Universities Osteoarthritis Index (WOMAC), were evaluated at 1 week, 4 weeks, 3 months, 6 months, 1 and 2 years after surgery. The length of the scar and the Scar Cosmesis Assessment and Rating (SCAR) scale were assessed at 1 year follow-up^19^. Patients’ satisfaction with the scar’s appearance (very satisfied, satisfied, or dissatisfied) was also recorded simultaneously.

### Secondary outcomes

**Figure 2:**
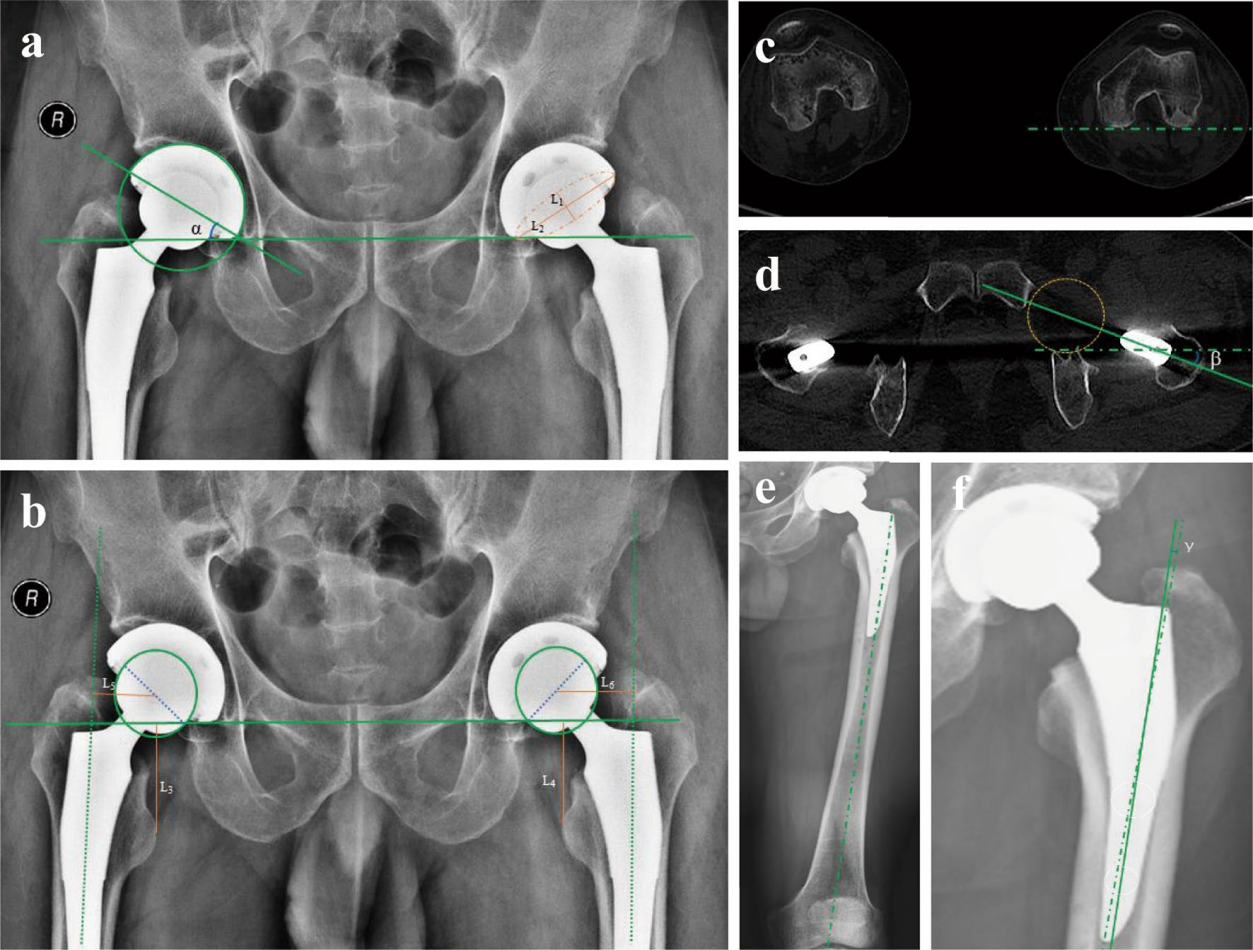
Radiographic measurements. (**a**) The methods of measuring the acetabular inclination angle (α) and acetabular anteversion angle on an AP radiograph, the long and short axes of the ellipse are used to calculate the acetabular anteversion angle, which is arcsin (L1/L2). (**b**) A line was drawn perpendicular to the inter-teardrop line and passed through the tip of the lesser trochanter on both sides (L3, L4). LLD was defined as the difference in distance between L3 and L4. Femoral offset was defined as the distance from the femoral head center to a line bisecting the long axis of the femur (L5, L6). (**c, d**) Femoral stem anteversion was defined as the angle (β) between the axis of the stem-neck and the posterior condylar axis of the femur. (**e, f**) Stem varus/valgus was measured as the angle (γ) between the anatomic axis of the femur and the axis of the femoral stem in the coronal plane. AP, anteroposterior; LLD, leg length discrepancy.

The operative time, including the procedural efficiency (defined by the procedure time) and operational efficiency (defined by the total time spent in the operating room (OR), were recorded^20^. Pelvic anteroposterior, standing full-length lower extremity radiographs, and full-length femoral CT scans were performed before and after the surgery. The acetabular inclination angle and acetabular anteversion angle were determined according to the measurement method described by Lin^21^ and Lewinnek et al.^22^ (Fig. 2a). Other radiographic measurements, including LLD **(**Fig. 2b), femoral offset (Fig. 2b), femoral anteversion (Fig. 2c,d), and stem varus/valgus angle (Fig. 2e,f), were measured according to our previous study^23^.

### Postoperative complications

We also analyzed the occurrence of adverse effects after surgery, including LFCN dysesthesia, intraoperative fracture, delayed wound healing, postoperative infection, dislocation, and deep venous thrombosis (DVT). Two independent investigators who did not participate in the surgery recorded all clinical and follow-up data.

### Statistical analysis

A pilot series was performed as a basis for power analysis. Assuming a power of 80%, a significance level of 0.05, and an expected loss to follow-up of 20% of the patients, we calculated a necessary total sample size of 96 patients. Statistical analysis was performed using SPSS 23.0 (IBM SPSS, Armonk, New York). Results are reported as mean ± SD. Pearson’s chi-squared test or Fisher’s Exact Test was used to analyze qualitative comparative data. Intergroup differences in radiographic measurements and continuous clinical data collected at each visit were assessed for significance using independent samples t-test after confirming that the data followed a normal distribution. When parameters were not normally distributed, the Mann–Whitney U-test was used.

## Results

A total of 106 patients were randomly assigned to the BI-DAA or the PLA group; each group of 50 patients was followed up for at least 2 years (Fig. 1). The baseline demographics and characteristics of the patients were summarized in Table 1, with no significant difference noted.

**Table 1:** Preoperative characteristics of patients.

Characteristic	PLA	BI-DAA	p-value
Age (years)	52.3 (12.6)	51.4 (13.6)	0.73^a^
Gender (%Female)	58	48	0.32^b^
BMI	21.9 (2.8)	21.8 (2.2)	0.84^a^
ASA Score	1.54 (0.50)	1.48 (0.50)	0.69^c^
Grade 1	23	26	
Grade 2	27	24	
Indications			
ONFH	21	22	
OA	26	22	
DDH (Crowe I/II)	3	6	
VAS	6.1 (1.2)	6.2 (0.9)	0.64^a^
HHS	49.2 (5.2)	49.8 (4.4)	0.53^a^
WOMAC	76.5 (6.8)	75.5 (5.8)	0.43^a^

Values are n (%) or mean (SD). P value < 0.05 considered statistically significant.

*BI-DAA* bikini-incision via direct anterior approach, *PLA* posterolateral approach, *BMI* body mass index, *ASA* American society of anesthesiologists, *ONFH* osteonecrosis of femoral head, *OA* osteoarthritis, *DDH* developmental dysplasia of the hip. *VAS* visual analogue scale, *HHS* harris hip score, *WOMAC* the western ontario and mcmaster universities osteoarthritis index, *SD* standard deviation.

^a^Student’s t test;

^b^Chi-squared test;

^c^Mann–Whitney U-test.

### Perioperative details

The patients in the BI-DAA group had a significantly shorter mean LOS than those in the PLA group (5.12 ± 1.5 vs. 6.40 ± 2.0 days, *P* < 0.01). The mean procedure time in the BI-DAA group was longer than in the PLA group (138.7 ± 17.8 vs. 130.6 ± 21.2 min, *P* = 0.04). However, compared with the PLA group, the BI-DAA group did not show a disadvantage in operational efficiency in terms of the total OR time (169.7 ± 17.3 vs. 167.5 ± 21.8 min, *P* = 0.58). The mean HGB drop (24.7 ± 13.3 g/L vs. 34.7 ± 16.7, *P* < 0.01) and the transfusion rate (9/50 vs. 18/50, *P* = 0.04) in the BI-DAA group were also significantly lower than that in the PLA group (Table 2).

**Table 2:** Perioperative outcomes in total hip arthroplasty.

Characteristic	PLA	BI-DAA	*p* value
Length of stay (day)	6.40 (2.0)	5.12 (1.5)	< 0.01^c^
Operating time (min)			
Procedure time	130.6 (21.2)	138.7 (17.8)	0.04^a^
Total OR time	167.5 (21.8)	169.7 (17.3)	0.58^a^
24 h HGB drop (g/L)	34.7 (16.7)	24.7 (13.3)	< 0.01^a^
Male	36.7 (17.2)	26.9 (13.5)	< 0.01^a^
Female	33.2 (16.5)	22.3 (12.9)	< 0.01^a^
Blood transfusion	18 (36)	9 (18)	0.04^b^

Values are n (%) or mean (SD). *p* value < 0.05 considered statistically significant.

*BI-DAA* bikini-incision via direct anterior approach, *PLA* posterolateral approach, *SD* standard deviation.

^a^Student’s t test;

^b^Chi-squared test;

^c^Mann-Whitney U-test.

### Radiographic analyses

**Figure 3:**
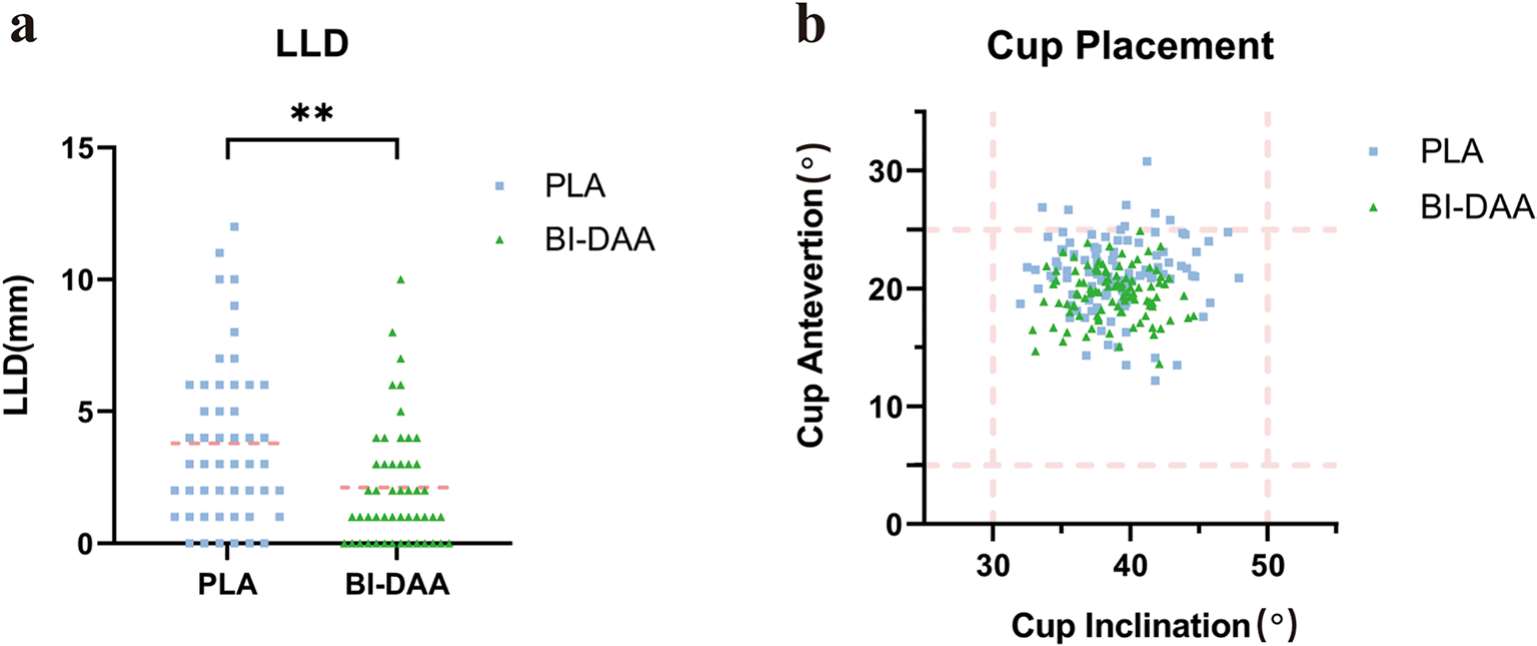
Prosthesis position evaluation. (**a**) The LLD value for each patient in the BI-DAA (green triangles) group and PLA group (blue squares), means of LLD in each group are noted as pink dot lines, **P < 0.01. (**b**) The scatter plot shows the acetabular inclination angles and anteversion angles of the BI-DAA group (green triangles) and PLA group (blue squares). The pink dot lines indicate the boundaries of the safe zones. BI-DAA, bikini-incision via direct anterior approach; PLA, posterolateral approach. LLD, leg length discrepancy.

The radiographic measurements are illustrated in Table 3. In brief, the mean LLD in the BI-DAA group was significantly smaller than that in the PLA group (2.1 ± 2.3 vs. 3.8 ± 3.0 mm, *P* < 0.01). Moreover, a LLD of ≤ 5 mm was found in 45 patients (45/50, 90%) in the BI-DAA group and only 36 patients (36/50, 72%) in the PLA group (*P* = 0.02) (Fig. 3a). There was no significant difference in the absolute difference in the femoral offset between the two groups (3.0 ± 2.5 vs. 3.4 ± 2.8 mm, *P* = 0.80). The acetabular inclination angle was not significantly different between the two groups (38.7 ± 2.6° vs. 39.1 ± 3.4°, *P* = 0.51). The average acetabular anteversion angle in the BI-DAA group was significantly smaller than that in the PLA group (18.6 ± 2.3° vs. 21.2 ± 3.2°, *P* < 0.01). All cups in the BI-DAA group (100/100, 100%) were placed in the safe zone, whereas 93 cups in the PLA group (93/100, 93%) were placed in the safe zone (*P* = 0.01) (Fig. 3b). In addition, the BI-DAA group had a smaller variance in cup inclination and anteversion (Fig. 3b). In terms of stem placement, there was no significant difference between the BI-DAA and PLA groups in stem anteversion (17.3 ± 1.9° vs. 17.4 ± 2.9°, *P* = 0.84) or the varus/valgus angle of the femoral stem (0.82 ± 0.4° vs. 0.79 ± 0.9°, *P* = 0.83).

**Table 3:** Radiographic measurements.

Characteristic	PLA	BI-DAA	*p* value
Absolute LLD (mm)	3.8 (3.0)	2.1 (2.3)	< 0.01^a^
≤ 5 mm	36 (72)	45 (90)	0.02^b^
Absolute difference of femoral offset (mm)	3.4 (2.8)	3.0 (2.5)	0.80^c^
Cup placement (°)			
Anteversion	21.2 (3.2)	18.6 (2.3)	< 0.01^c^
Inclination	39.1 (3.4)	38.7 (2.6)	0.51^a^
Stem placement (°)			
Anteversion	17.4 (2.9)	17.3 (1.9)	0.84^a^
Varus/Valgus	0.79 (0.9)	0.82 (0.4)	0.83^a^

Values are n (%) or mean (SD). *p* value < 0.05 considered statistically significant.

*LLD* leg length discrepancy; *SD* standard deviation.

^a^Student’s t test;

^b^Chi-squared test;

^c^Mann–Whitney U-test.

### Pain and functional outcomes

The postoperative pain and functional outcomes up to the 2 year follow-up are shown in Table 4. In brief, the VAS scores in the BI-DAA group evaluated within 7 days after the operation were better than those in the PLA group (*P* ≤ 0.01). However, the difference diminished afterward. Overall, the BI-DAA group tended to have a higher HHS than did the PLA group at 7 days (82.1 ± 3.9 vs. 75.8 ± 6.7, *P* < 0.01), 4 weeks (90.0 ± 2.9 vs. 85.6 ± 3.1, *P* < 0.01) and 3 months postoperatively (92.5 ± 2.3 vs. 91.6 ± 1.8, *P* = 0.03). The WOMAC scores at 1 week (19.1 ± 3.5 vs. 21.4 ± 4.8, *P* < 0.01), 4 weeks (14.0 ± 3.0 vs. 16.7 ± 2.7, *P* < 0.01), and 3 months (4.8 ± 1.9 vs. 6.5 ± 2.2, *P* < 0.01) postoperatively showed that the BI-DAA group continued to have better results than the PLA group for 3 months after surgery. However, the BI-DAA group's differences (in the HHS and WOMAC scores) were no longer evident at the 6-month follow-up and after that.

**Table 4:** Postoperative pain and functional outcomes.

Variable (Mean ± SD)	VAS			HHS			WOMAC		
	PLA	BI-DAA	*p* value	PLA	BI-DAA	*p* value	PLA	BI-DAA	*p* value
Time point									
24 h	3.7 (0.8)	3.2 (1.1)	0.01^a^	NA	NA	NA	NA	NA	NA
72 h	3.0 (0.8)	2.2 (0.9)	< 0.01^a^	NA	NA	NA	NA	NA	NA
1 w	2.4 (0.8)	1.8 (0.6)	< 0.01^a^	75.8 (6.7)	82.1 (3.9)	< 0.01^a^	21.4 (4.8)	19.1 (3.5)	< 0.01^a^
4 w	1.7 (0.5)	1.6 (0.5)	0.32^a^	85.6 (3.1)	90.0 (2.9)	< 0.01^a^	16.7 (2.7)	14.0 (3.0)	< 0.01^a^
3 m	1.5 (0.5)	1.4 (0.5)	0.32^a^	91.6 (1.8)	92.5 (2.3)	0.03^a^	6.5 (2.2)	4.8 (1.9)	< 0.01^a^
6 m	NA	NA	NA	94.2 (1.9)	94.8 (2.5)	0.18^a^	4.9 (2.1)	4.5 (2.0)	0.33^a^
12 m	NA	NA	NA	94.8 (1.8)	95.0 (2.1)	0.61^a^	4.5 (2.3)	4.3 (2.2)	0.66^a^
24 m	NA	NA	NA	95.1 (2.0)	95.3 (1.8)	0.60^a^	4.3 (1.8)	4.2 (1.8)	0.78^a^

Values are mean (SD). *p* value < 0.05 considered statistically significant.

*VAS* visual analogue scale, *HHS* harris hip score, *WOMAC* the western ontario and mcmaster universities osteoarthritis index, *SD* standard deviation.

^a^Student’s t test.

### Scars and complications

Compared to the PLA group, the patients who underwent the BI-DAA had significantly shorter scars (9.7 ± 1.6 vs. 10.8 ± 2.0 mm, *P* < 0.01), lower SCAR scores (4.2 ± 0.9 vs. 6.7 ± 0.9, *P* < 0.01), and higher satisfaction with the scars, based on a follow-up of the wounds performed one year after surgery (Table 5). Patients in the BI-DAA group tended to show a higher incidence of LFCN dysesthesia (12/100 vs. 0/100 thighs, *P* < 0.01). Five patients in the BI-DAA group still reported numbness in the region innervated by LFCN at 1 year follow-up. There was no difference (*P* > 0.99) in other postoperative adverse effects, including wound complications, dislocation, intraoperative fracture, venous thromboembolism, or postoperative infection. No patients returned to the hospital or underwent revision surgery during the follow-up period.

**Table 5:** Post-Operation Scar and Wound Evaluation.

Characteristic	PLA	BI-DAA	*p* value
Length of scar (mm)	10.8 (2.0)	9.7 (1.6)	< .01^a^
SCAR score	6.7 (0.9)	4.2 (0.9)	< .01^b^
Satisfaction with scar			
Not satisfied	3 (6%)	2 (4%)	
Satisfied	28(56%)	11 (22%)	
Very satisfied	19 (38%)	37 (74%)	

Values are n (%) or mean (SD). *p* value < 0.05 considered statistically significant.

*BI-DAA* bikini incision via direct anterior approach, *PLA* posterolateral approach, *SCAR scar cosmesis assessment, and rating*. *SD* standard deviation.

^a^Student’s t test,

^b^Mann-Whitney U-test.

## Discussion

Currently, although simBTHA is an effective treatment for an end-stage bilateral hip disease, few prospective studies have been designed to focused on strategies to reduce its perioperative burden and accelerate postoperative recovery^24,25^. A prospective randomized controlled trial was conducted to compare perioperative and early postoperative outcomes between the bikini incision and the PLA among simBTHA patients and we hypothesized that bikini incision could enhance the early recovery, the accuracy of prosthesis orientation, early postoperative outcomes, and scar healing. Interest in surgical approach to THA continues to be high. Specific pros and cons of the DAA vs PLA have been debated in the literature with no clear consensus^9,10^. This study compared the early outcomes of two different surgical procedures after simBTHA. It showed that the bikini incision has certain strategic advantages over the PLA in shortening LOS, improving early postoperative recovery, controlling LLD, improving component implantation accuracy, and scar satisfaction rates.

We found the bikini incision associated with a smaller HGB drop and a lower transfusion requirement than the PLA in the early postoperative period. One of the important reasons is that bikini incision utilizes the neuromuscular interval and has the advantages of a shorter invasive incision, reduced soft tissue damage, and intraoperative bleeding. The BI-DAA group also favoured LOS, early VAS, and functional scores after simBTHA surgery. We found that patients treated with bikini incision THA had a shorter LOS than those treated with PLA THA, consistent with the findings of previous unilateral studies^26,27^. Previous unilateral studies have concluded that the bikini incision takes more time to perform than the PLA due to narrow access and a poor visual field^28^. However, in this study, we found that the difference between the two groups in total OR time was not evident because the surgeons in the BI-DAA group were able to start the surgery on the contralateral side immediately after the main procedure on the first side due to the supine position. Although PLA with lateral decubitus takes more time to install the patient than a DAA in supine position on a conventional table. For an experienced surgeon, the surgical time should be lower in the BI-DAA group among simBTHA patients^13^. In an RCT involving 120 patients and comparing early functional recovery after unilateral THA via the DAA or PLA, DAA-THA was found to be associated with higher HHS and UCLA activity scores at 3 months postoperatively than was the PLA, showing that the bikini incision yields better early pain relief and postoperative functional recovery after simBTHA than does the PLA^13,29,30^. In addition, a prospective study showed that functional recovery as measured by the timed up-and-go (TUG) test and the motor component of the Functional Independence Measure™ (M-FIM™) occurred up to 2 weeks sooner in patients treated by the DAA than in those treated by the PLA^30^. However, previous studies did not suggest that the bikini incision produced better long-term outcomes than the PLA in activity recovery^13,29^. Our study is consistent with the previous studies and found that the difference in functional scores disappeared in a short period (6 months), which may be partially attributable to the bilateral surgery.

Two of the most common problems with the classic DAA are low patients’ subjective scar satisfaction and disastrous postoperative wound complications^17,28^. The wound-related outcomes in this study were in line with our expectations. The bikini-incision method yields a shorter length of the scar, better wound healing, and higher satisfaction due to the alignment of the oblique incision with the direction of the anatomical skin tension line. Moreover, our results showed that patients who undergo the bikini incision bilaterally have smaller variability in component placement. LLD is one of the most common complications after THA and may lead to nerve damage, gait disturbances, and low back pain^31–33^. We believe that the bikini incision yields better control of the LLD because the patient is in a supine position, allowing the surgeon to compare the leg length more easily and accurately than during the PLA. In this trail, intraoperative fluoroscopy and a freehand technique were used to ensure the equality of lower limbs length. Our findings are supported by a meta-analysis with a total of 34,873 patients^34^; that analysis found that LLD was smaller in the DAA group than the PLA group. The differences within groups in the radiographic results were smaller for the BI-DAA group than the PLA group, suggesting that the bikini incision yields less variability in the acetabular anteversion and inclination angles. Similar to our findings, a previous RCT that compared cup placement between the DAA and PLA in 120 patients not only found that the DAA group had smaller variability in component position than did the PLA group^13^.

LFCN injuries are widely recognized as severe complications of DAA surgery^37^. In our study, 12% of the thighs in the BI-DAA group had decompensated anterior thigh anaesthesia, which has been reported to have a moderate incidence in previous studies, ranging from 15 to 81%^37–39^. Furthermore, we believe that the sensory abnormalities are mainly associated with reversible LFCN injuries, which may be due to reversible peeling injury caused by intraoperative traction of the retractor, as 80% patients experienced relief of their numbness symptoms during the follow-up period, which is consistent with previous study conducted by Ozaki^40^. Another concern about the bikini incision is the steep learning curve. Without specific training and experience, these favourable results may not only be immediately reproducible but lead to poor postoperative recovery and numerous postoperative complications^41–43^.

The present design is not without limitations. First, in our study, all procedures were performed by a single surgeon. Although bias between the two groups stemming from surgical skills can be eliminated, our results should be validated and extended in further research that includes a broader range of surgeons. Second, the investigator assessing postoperative outcomes was not blinded to the group assignments, which may create bias in the study. Third, the functional aspect is evaluated only by the WOMAC and HHS in this study. It is necessary to take disorders of static and dynamic posture (gait analysis, stabilometry), functional tests into consideration to get a full view of the functional aspect after surgery. Fourth, we did not analyze outcomes or complications beyond two years after surgery. Further mid-term and long-term data regarding complication rate and implant survivorship are needed to allow us to fully assess the safety and necessity of the bikini incision in simBTHA.

In conclusion, our study illustrates that the bikini incision can be performed safely with few complications and yield a shorter LOS, a lower blood transfusion rate, faster early functional recovery, better scar recovery and may have potential effects in components placement than the PLA in simBTHA. Therefore, we believe the bikini incision could be a feasible and safe option for patients undergoing single-stage BTHA.

## Data Availability

The datasets generated and analyzed during the current study are not publicly available since this was not a part of the informed consent signed by the participants, but are available from the corresponding author on reasonable request.
